# Next-generation pyrosequencing of gonad transcriptomes in the polyploid lake sturgeon (*Acipenser fulvescens*): the relative merits of normalization and rarefaction in gene discovery

**DOI:** 10.1186/1471-2164-10-203

**Published:** 2009-04-29

**Authors:** Matthew C Hale, Cory R McCormick, James R Jackson, J Andrew DeWoody

**Affiliations:** 1 Department of Forestry and Natural Resources, Purdue University, West Lafayette, IN 47907, USA; 2 Cornell Biological Field Station, 900 Shackelton Point Road, Bridgeport, NY 13030, USA

## Abstract

**Background:**

Next-generation sequencing technologies have been applied most often to model organisms or species closely related to a model. However, these methods have the potential to be valuable in many wild organisms, including those of conservation concern. We used Roche 454 pyrosequencing to characterize gene expression in polyploid lake sturgeon (*Acipenser fulvescens*) gonads.

**Results:**

Titration runs on a Roche 454 GS-FLX produced more than 47,000 sequencing reads. These reads represented 20,741 unique sequences that passed quality control (mean length = 186 bp). These were assembled into 1,831 contigs (mean contig depth = 4.1 sequences). Over 4,000 sequencing reads (~19%) were assigned gene ontologies, mostly to protein, RNA, and ion binding. A total of 877 candidate SNPs were identified from > 50 different genes. We employed an analytical approach from theoretical ecology (rarefaction) to evaluate depth of sequencing coverage relative to gene discovery. We also considered the relative merits of normalized versus native cDNA libraries when using next-generation sequencing platforms. Not surprisingly, fewer genes from the normalized libraries were rRNA subunits. Rarefaction suggests that normalization has little influence on the efficiency of gene discovery, at least when working with thousands of reads from a single tissue type.

**Conclusion:**

Our data indicate that titration runs on 454 sequencers can characterize thousands of expressed sequence tags which can be used to identify SNPs, gene ontologies, and levels of gene expression in species of conservation concern. We anticipate that rarefaction will be useful in evaluations of gene discovery and that next-generation sequencing technologies hold great potential for the study of other non-model organisms.

## Background

Massively parallel pyrosequencing via Roche's 454 platform [1] has great potential for identifying genes of interest to ecologists and evolutionary biologists. The 454 approach provides more accurate base calling and deeper sequencing coverage than is possible with conventional Sanger sequencing while dramatically decreasing labor [1-3]. For the most part, pyrosequencing has so far been restricted to model organisms [3-6] or species closely related to a model [7] because of the short reads which make *de novo *genome sequencing difficult without a scaffold [8]. However, the depth of coverage provided by the Roche 454 platform means that transcriptomes of non-model organisms can be characterized without a genome sequence. Recently, 454 technology has been applied to transcriptomes of the rose gum tree (*Eucalyptus grandis *[9]) and the Glanville fritillary butterfly (*Melitaea cinxia *[10]). These studies illustrate the potential of 454 pyrosequencing to rapidly characterize expressed genes that can be used to address pertinent questions regarding a species' ecology, life history, and evolution [11,12].

A complete description of expressed sequence tags (ESTs) provides an overview of the transcriptome, those genes expressed (transcribed) in a given tissue at a specific point in time. Pyrosequencing of ESTs can be used to characterize gene expression [6], QTL [13], single nucleotide polymorphisms (SNPs; [9,14]), and patterns of selection [9]. The identification of SNPs is especially appealing in non-model species because as genetic markers, SNPs can illuminate population structure, sex ratios, and genetic variability [15]. Recently, 454 sequencing has been used to identify tens of thousands of SNPs in inbred lines of maize and in *E. grandis *[14,9] demonstrating the power of pyrosequencing for SNP discovery.

There are many factors to be considered prior to transcriptome characterization, including the expected number of unique transcripts in the sampled tissue(s) and their relative abundance. When it comes to sequencing effort, transcriptome sequencing needs to be deep in order to identify rare transcripts and to overcome short read lengths, but the absolute depth required is usually unknown although important for quantifying levels of gene expression. If the relative abundance of transcripts is not critical, normalization can enhance the identification of rare transcripts by reducing the number of overabundant transcripts [16]. This is especially important when sequencing from cDNA pooled from many different organs/tissues or individuals (e.g. [7,9]). However, in non-model species with poorly characterized genomes, it may not be apparent if relative gene expression is responsible for an interesting phenotype. To date, the costs and benefits of normalization have been evaluated only in model species where entire genome sequences are available (e.g. *Arabidopsis*; [3]).

We are interested in the sex determining mechanisms of fishes, and 454 pyrosequencing of gonad transcriptomes may provide a powerful approach for understanding the genetic architecture of sexual differentiation. In particular, we are interested in lake sturgeon (*Acipenser fulvescens*), a species of conservation concern in North America due to historical overharvest, pollution and habitat fragmentation [17]. The restoration of lake sturgeon populations is complicated by their biology, namely delayed sexual maturity (between 10 and 30 years of age), infrequent spawning (every few years), and sexual monomorphism [18]. With regard to the latter issue, DNA sexing assays have proven invaluable in the conservation of other sexually monomorphic species and could be a great boon for lake sturgeon biologists [19]. However, the search for sturgeon sex determining genes has so far been unsuccessful [18,20,21]. This is probably because the genetics of sex determination in fishes is much more complicated (i.e., evolutionary labile) than in mammals and birds [19,22,23]. Furthermore, the lake sturgeon genome is large (5 times the size of humans) and polyploid [24].

Herein we present the results of a pilot study designed to characterize gonad transcriptomes in lake sturgeon. To our knowledge, this is the first such study in vertebrates and the first in a polyploid species. Our ultimate goal is to provide an overview of transcription in lake sturgeon gonads, including the discovery of new genes and the SNPs they harbor. Our proximate goals were a) to evaluate the relative merits of normalization for 454 runs and b) to evaluate an ecological technique used to determine species richness (rarefaction) and establish its usefulness in gene discovery. We did so by performing five titration runs on the 454 using two normalized and three non-normalized (hereafter referred to as native) pools of cDNA.

## Results

### Gene and SNP discovery

#### Sequence assembly

**Figure 1:**
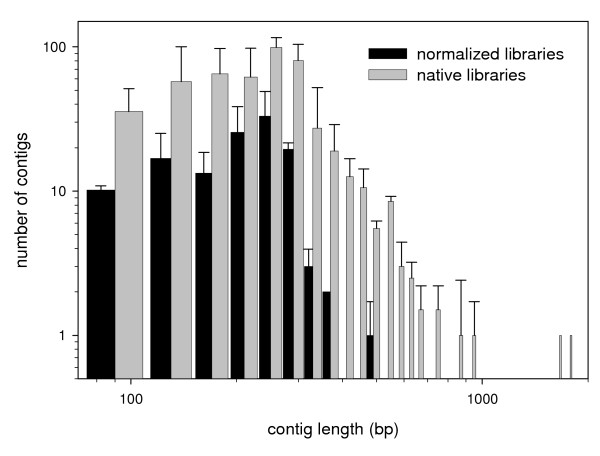
**Length of contigs as averaged (± SD) between the normalized and native libraries**. Note the logarithmic axes.

**Figure 2:**
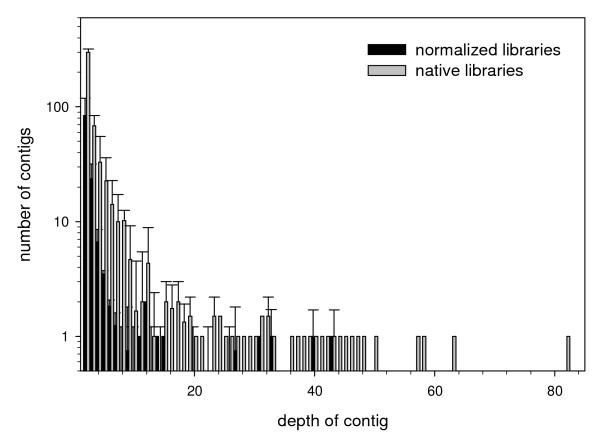
**Depth of contigs as averaged (± SD) between the normalized and native libraries**.

Libraries 1 and 2 were normalized whereas native libraries 3–5 were not. The titration runs produced 3,811, 13,414, 11,178, 10,296 and 8,361 sequences from libraries one through five respectively, for a total of 47,060 reads. These yielded 1,234, 1,385, 8,700, 5,061 and 4,361 reads after quality control (a total of 20,741; Table 1). Mean read length varied across libraries, with longer sequences in the normalized libraries (range 143–232 bp; Table 1; Figure 1). The number of contigs varied from 110 to 578 across libraries and mean read depth per contig varied from 2.8 to 4.9 sequences (Table 2; Figure 2). Figures 1 and 2 represent contig length and contig depth as averaged across the normalized and native libraries. Regression analysis indicated there was a significant positive relationship between contig length and contig depth (r^2 ^= 0.25, slope = 5.861 (± 0.237), p = < 0.001).

**Table 1:** Number of reads and nucleotides produced by five cDNA libraries (L1–L5). Significant BLAST hit criteria were an e-value ≤ 1 × 10^-3 ^and a bit score > 40.

	L1	L2	L3	L4	L5	Total
Tissue source	adolescent male	adolescent female	unk. juvenile	adolescent male	adolescent female	n/a
Status	normalized	normalized	native	native	native	n/a
N. reads	3,811	13,414	11,178	10,296	8,361	47,060
N. reads after trimming	1,234	1,385	8,700	5,061	4,361	20,741
N. nucleotides	286,822	270,099	1,247,400	870,023	869,912	3,544,256
Mean read length	232	195	143	162	199	186
Median read length	251	218	133	170	231	201
Mode read length	243	261	100	75	240	184
Significant BLAST hits	241	262	1,926	996	1,585	5,010

**Table 2:** Contig summary statistics from the PCAP analyses of all five libraries (L1–L5).

	L1	L2	L3	L4	L5
number of contigs	170	110	572	578	401
mean depth of contig	2.8	3.7	4.5	4.3	4.9
mean length of contig	197	200	187	232	274
number of reads in contigs	481	397	2,578	3,041	2,374
number of singletons	753	988	6,122	7,834	2,390

#### Xenobiotics

**Figure 3:**
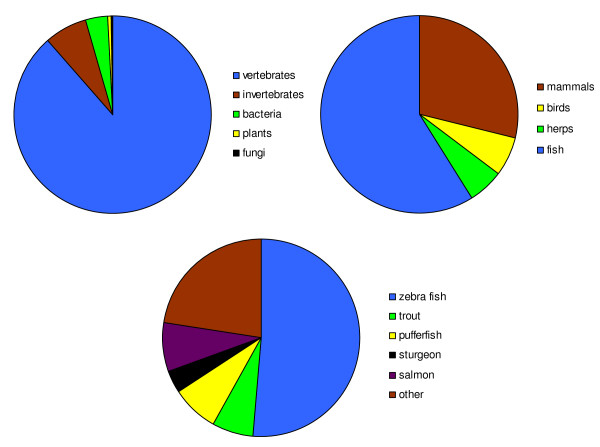
**Taxonomic distribution of the BLAST results**. (a) The percentage of BLAST hits that are vertebrate, invertebrate, bacteria, plant, and fungi. (b) The percentage of vertebrate blast hits that are mammals, birds, herps, and fish. (c) The percentage of BLAST fish hits that are zebrafish, trout, puffer fish, sole, flounder, sturgeon, and other fish species.

Pyrosequencing from metazoan tissues can produce sequences from endosymbionts [10]. Of the lake sturgeon ESTs that passed quality control (QC), 88.7% blasted back to vertebrates, 6.9% to invertebrates, 3.7% to bacteria, 0.4% to plants, and 0.3% to fungi (Figure 3a). The mean e-value for the top BLAST hits to vertebrates was 9.9 × 10^-05 ^(± 0.001), compared to 8.4 × 10^-04 ^(± 0.002) for non-vertebrate taxa. Of the ESTs with a top BLAST hit to vertebrates, 59% had a top BLAST hit to a fish species, 29% matched a mammal, 5.9% a reptile or amphibian, and 6.2% a bird (Figure 3b). The BLAST hits to fishes most frequently matched zebrafish, pufferfish, and trout (Figure 3c).

#### Gene ontologies

All lake sturgeon reads that passed QC were annotated for sequence similarities using BLASTx against the GenBank database; over 5,000 produced a significant BLASTx hit (Table 1). Gene Ontology (GO) assignments were broken into Molecular Function, Biological Process, and Cellular Components. GO analyses categorized ESTs into one of 115 Molecular Function categories; those most highly represented include protein binding, RNA binding, and zinc ion binding. Sixteen of the 115 Molecular Function categories (13.9%) differed statistically with regard to their representation between normalized and native libraries (Table 3). For example, a much higher proportion of sequences from the native libraries were RNA binding genes (18.3%) than in the normalized libraries (5.3%; P-value = 0.001). A higher proportion of sequences from the normalized libraries were involved in several key pathways (such as ATP binding, oxygen binding, and zinc ion binding) than in the native libraries (see Additional files 1, 2 and 3).

**Table 3:** Of 115 different Molecular Functions identified in the Gene Ontology analysis, 16 differed significantly in expression between normalized and native libraries.

Molecular Function	Total counts normalized	% of counts	Total counts native	% of counts	P-value
actin binding	7	1.13	24	0.30	*
ATP binding	101	16.29	152	1.88	***
cytochrome c oxidase	78	12.58	155	1.92	***
DNA binding	23	3.71	76	0.94	***
GTP binding	6	0.97	22	0.27	*
isomerase activity	4	0.65	8	0.10	**
kinase activity	4	0.65	5	0.06	**
motor activity	4	0.65	0	0.00	***
oxygen binding	12	1.94	2	0.02	***
protein binding	178	28.71	1348	16.71	**
ribosome biogenesis	0	0.00	811	10.05	***
RNA binding	33	5.32	1482	18.37	***
structural constituent of ribosome	0	0.00	1791	22.20	***
sugar binding	2	0.32	6	0.07	**
ubiquinol-cytochrome-c reductase activity	22	3.55	6	0.07	***
zinc ion binding	136	21.94	725	8.99	***

P-value significance; * = 0.05, ** = 0.01, *** = 0.001.

GO analysis identified 66 Biological Process categories, including translation, muscle cell differentiation, and transport. Fifteen of these 66 (22.7%) were represented unequally between the normalized and native libraries (Table 4). Not surprisingly, genes involved in protein translation exhibited the biggest differences in expression; 12.8% of sequences in the normalized libraries were involved in translation compared to 40.7% of sequences in the native libraries (p < 0.001).

**Table 4:** List of genes found within the Biological Process category of the Gene Ontology assignment with a significant difference in expression between normalized and native libraries.

Biological Process	Total counts normalized libraries	% counts	Total counts native libraries	% counts	P-value
apoptosis	6	2.08	8	0.18	***
biosynthetic process	14	4.84	2	0.04	***
cell adhesion	8	2.77	11	0.24	***
DNA repair	14	4.84	9	0.2	***
glycoloysis	7	2.42	8	0.18	***
immune response	7	2.42	6	0.13	***
metabolic process	26	9.0	4	0.09	***
muscle cell differentiation	95	32.9	4	0.09	***
positive regulation of apoptosis	3	1.04	6	0.13	**
protein folding	15	5.19	25	0.56	***
protein transport	4	1.38	17	0.38	**
proteolysis	6	2.08	21	0.47	**
regulation of cell shape	0	0	610	13.55	***
translation	37	12.80	1830	40.66	**
transport	40	13.84	211	4.69	***

P-value significance; * = 0.05, ** = 0.01, *** = 0.001.

GO analysis identified 62 Cellular Components among our ESTs, including cytoplasm, mitochondrion, and membrane. Twelve of the 62 (19.3%) were represented unequally between the libraries (Table 5). Four gene classes (small cytosolic subunit, large cytosolic subunit, plasma membrane and ribosome) were overrepresented in the native libraries. The other 8 were more abundant in the normalized libraries.

**Table 5:** List of genes found within the Cellular Component category of the Gene Ontology assignment with a significant difference in expression between normalized and native libraries.

Cellular Component	Total counts normalized libraries	% counts	Total counts native libraries	% counts	P-value
actin filament	93	12.77	24	0.65	***
cytoplasm	187	25.69	225	6.10	***
cytosol	46	6.32	42	1.14	***
cytosolic large ribosomal subunit	0	0.00	193	5.23	**
cytosolic small subunit	0	0.00	810	21.97	***
ER	16	2.20	43	1.17	*
golgi apparatus	9	1.24	20	0.54	*
membrane	110	15.11	239	6.48	***
mitochrondrion	111	15.25	66	1.79	***
perinuclear region of cytoplasm	15	2.06	19	0.52	***
plasma membrane	4	0.55	207	5.61	**
ribosome	25	3.43	645	17.49	***

P-value significance; * = 0.05, ** = 0.01, *** = 0.001.

#### SNP detection

**Figure 4:**
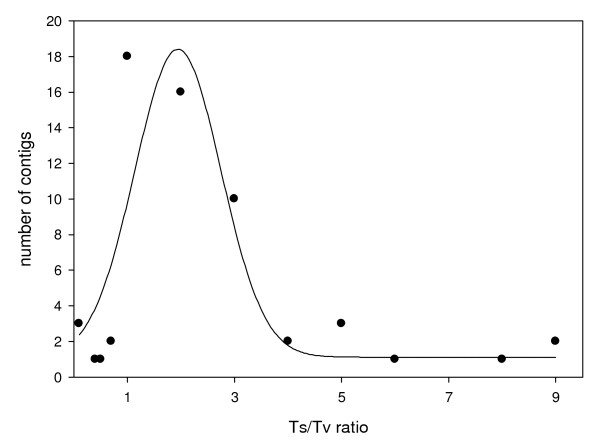
**Frequency plot of Ts/Tv ratios for 60 contigs with a significant BLAST hit**. Points represent the number of contigs with that specific Ts/Tv ratio. Ts/Tv ratios greater than 1 indicate an excess of transitions, those lower than 1 indicate an excess of transversions. A line of best fit is plotted through the data.

We identified 877 candidate SNPs from 1,840 contigs whose sequences spanned 403,258 bp; this equates to 1 SNP every 460 bp. Of the 877 SNPs, only 16 were insertion/deletions whereas 861 were substitutions. A total of 722 SNPs (82.3%) were singletons present in one read within a contig. Of the 155 SNPs that appeared in multiple reads, 66 occurred in different fish (libraries). Table 6 summarizes a subset of the SNP data, those SNPs found in genes of known function, and with a Ts/Tv ratio of less than 1 (more transversions than transitions) these genes could be targets of selection (see Additional file 4 for all SNP data). SNP density varied across genes, this may be due in part to strong historical selection, and the Ts/Tv ratio can help identify such genes [25,26]. A total of 561 SNPs were transitions and 300 SNPs were transversions, giving a mean Ts/Tv ratio of 1.87 across the transcriptomes. We estimated the Ts/Tv ratio for all contigs that BLASTed to a known gene (see Additional file 5); their frequency distribution is shown in Figure 4.

**Table 6:** Subset of those contigs constructed from all five cDNA libraries combined (20,741 individual reads).

Contig ID	Length	Depth	# SNPs	bp per SNP	Ts/Tv ratio	Top BLAST hit	GENBANK ID	Bit score	e-value
Contig18.1	421	28	5	84.2	0.7	CXXC finger 6	XP_001072947.1	53	7.00E-06
Contig19.1	379	27	8	47.4	0.5	Ribosomal S20	XP_001514106.1	181	2.00E-44
Contig26.1	630	24	2	315.0	0.0	ORF2	AAC60281.1	89	1.00E-22
Contig61.1	465	13	1	465.0	0.0	Calmodulin Complex	2VAY	202	7.00E-51
Contig65.1	553	13	7	79.0	0.8	Endonuclease-reverse transcriptase	XP_001182458.1	80	6.00E-14
Contig176.1	311	7	14	22.2	0.4	Polyprotein	AAN12398.1	89	1.00E-16
Contig278.1	485	5	3	161.7	0.0	Tc1-like transporase	BAF37936.1	121	2.00E-26

The contigs listed below include only those with a significant BLAST hit **and **at least one SNP **and **with a Ts/Tv ratio < 1.0 (see Methods). In other words, the SNPs described in this table are a subset of the 877 we identified, but includes all of those associated with a particular gene and with a Ts/Tv ratio < 1.0. See additional file 5 for information from those contigs that produced a Ts/Tv ratio ≥ 1.0.

### Rarefaction and normalization

**Figure 5:**
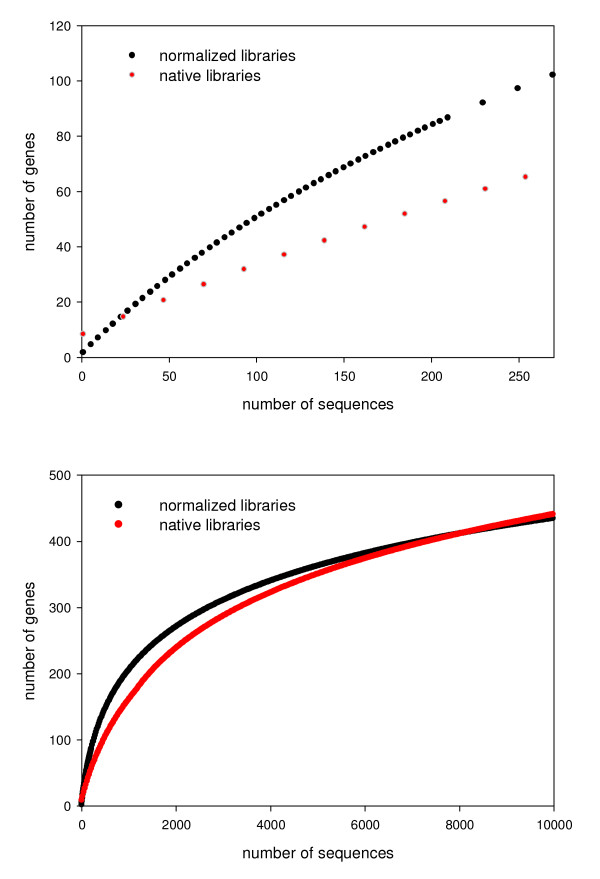
**Rarefaction curves illustrating gene discovery (number of genes) as a function of effort (number of sequencing reads)**. (a) Rarefaction analysis comparing empirical gene discovery between normalized and native libraries. (b) Rarefaction analysis comparing simulated gene discovery between normalized and native libraries.

Rarefaction analysis was pioneered in theoretical ecology, where it was used to evaluate species richness [27,28]. It has more recently been adopted in population genetics [29-31] and it has potential utility in genomics, particularly with regard to gene discovery as a function of effort. Figure 5 illustrates the difference in gene discovery rates in normalized and native libraries. Figure 5a plots our empirical data with the curves truncated at 260 reads (the number present in the normalized libraries), whereas Figure 5b shows simulated data based on the projection of the curves in Figure 5a. When using both empirical and simulated data, the rate of gene discovery is moderately faster in the normalized libraries, but only up to ~4,000 sequences.

## Discussion

### Gene and SNP discovery

#### Sequence assembly

We performed five Roche 454 titration runs on lake sturgeon cDNA samples with the ultimate goal of characterizing (for the first time) a polyploid vertebrate transcriptome. The lake sturgeon data compare favorably with other 454 cDNA studies of non-model species, both in terms of read length and contig assembly. Mean length of lake sturgeon reads after removing primers, linkers, and substandard bases was 190 bp, in between the 111 bp and 209 bp reported in similar non-model 454 studies [9,10]. Likewise, our mean contig length (218 bp) and depth (4.1 reads) were similar to the fritillary assembly (197 bp and 2.9 reads) and the Eucalypt assembly (353 bp and 9.9 sequences). Reads generated by 454 sequencing are generally shorter than those produced by dideoxy sequencing, but the base-calling accuracy of the 454 is equivalent or even superior to conventional sequencing [1]. Furthermore, the absolute number of reads produced in a run can help increase overall accuracy.

#### Xenobiotics

In the Glanville Fritillary butterfly, 454 transcriptomics provided evidence of microsporidia, intracellular parasites that affect insect population dynamics [32,10]. Thus it is possible that some of the sequences in our data set are from xenobiotics that are commensal with lake sturgeon. However, 88.7% of our sequences with a significant BLAST hit best matched a vertebrate, and of these 59% best matched another fish suggesting the vast majority of our sequences originated from lake sturgeon. Interestingly, 156 (3.7%) of our sequences BLASTed to a bacterial species. This could be due to noise (i.e., the lack of closely related sequences in GenBank) or to true xenobiotics that are lake sturgeon commensals, pathogens, or parasites. Of these 156 sequences, the average top BLAST hit had an e-value of 1.6 × 10^-04^, an order of magnitude less than the average top BLAST hits to vertebrates (e-value = 9.9 × 10^-05^) but still high, suggesting the presence of xenobiotics in our gonad samples. The top BLAST hits to bacteria were from a variety of species, of which 6.4% were aquatic. Of the reads that BLASTed to bacteria, 14.7% were to pathogenic species. Because our sequences were derived from reproductive tissues, some of the bacterial sequences could belong to sexually transmitted pathogens or *Wolbachia*-type microorganisms, although our preliminary analyses revealed no hits to sexually transmitted microorganisms. Further investigation of lake sturgeon xenobiotics could prove informative regarding coevolution, disease, and infection.

#### Gene ontologies

In principle, GO analysis can help gauge the merits and demerits of normalization. By comparing GO results from the normalized and native libraries, some interesting trends emerge. For instance, 32% of the annotated genes within the GO Molecular Function category were singletons in the native libraries compared to 6% in the normalized libraries. [These numbers include only those reads that produced a significant BLAST hit; due to the lack of related sequences in Genbank, most (74%) reads did not produce a significant BLAST hit.] Results from the Biological Process category were similar, with singletons comprising 32% of the native libraries compared to 15% in the normalized libraries. In terms of Molecular Function, the most striking result is the abundance of rRNA genes (as shown by the terms "Ribosome biogenesis" and "RNA binding" in Table 3). A total of 18.2% of all sequences from the native libraries are RNA binding genes as compared to 5.3% from the normalized libraries. In other words, normalization is effective at tempering the signature of abundant transcripts exactly as expected. However, even without normalization more than 60% of transcripts were not rRNAs. This suggests that many transcriptomics studies should forego normalization, as native libraries allow one to answer questions regarding gene dosage (i.e., relative expression levels) whereas normalized libraries do not. Clearly, this decision depends on the depth of coverage expected and the design of the study.

#### SNP detection

We identified a total of 877 candidate SNPs in lake sturgeon, about one every 460 bp. This compares to one in every 192 bp in Eucalypt [9] and one in 214 bp in maize [14]. Previous studies have used Sanger sequencing to confirm ~80% of SNPs initially identified via 454 pyrosequencing [9]. Due to the polyploid nature of the lake sturgeon genome, we might have expected more diversity than in the diploids (because of duplicate genes), but as mentioned earlier lake sturgeon are of conservation concern. Our source population was established from a few wild fish less than 20 years ago [33], and thus our SNP data may not reflect broader patterns of genetic diversity in lake sturgeon. The SNP frequency estimate of 1 per ~460 bp includes all SNPs identified in all contigs in the data set. However, the SNP frequency in contigs that produced a significant BLAST hit was 1 per ~142 bp. This is due to the removal of short contigs that are less likely to both a) contain SNPs and b) produce a significant BLAST hit. This bias is especially prevalent in our data as the majority of contigs are ≤ 300 bps.

Our SNP discovery requirements were stringent (see Methods) and our estimates of SNP density should be quite conservative. For example, we documented only 16 indels despite the susceptibility of 454 sequencing technology to indel-type errors [1]. Sixty-six of the candidate SNPs we identified were seen in multiple fish; these could be orthologous intraspecific polymorphisms (useful for population genetic analyses) or intragenomic polymorphisms due to gene duplications (useful for reconstructing gene trees). Population surveys will be required to discriminate among these alternatives. Eighteen candidate SNPs were unique to one sex; 16 were unique to males (i.e., found in multiple male libraries but not in female libraries) and 2 unique to female (i.e., found in both female libraries but not in male libraries). Three contigs that had SNPs unique to males produced a significant BLAST hit; contigs 27.1, 58.1 and 258.1, which produced a top BLAST hit to *ORF1*, *transposase*, and a muscle binding protein respectively. None of these genes are expected *a priori *to be involved in sexual differentiation, but further research will determine if these candidate SNPs might play a role in sex determination or will prove useful for sex identification.

We investigated the Ts/Tv ratio of SNPs in genes with a significant BLAST hit to help identify potential targets of selection, as transversions are rarer than transitions in neutral genes [25]. The plot of Ts/Tv ratios (Figure 4) shows that the majority of genes have either a Ts/Tv ratio near 1 (equal number of transitions and transversions) or greater than 1 (more transitions than transversions). Only seven contigs produced a Ts/Tv ratio < 1 (more transversions than transitions). These contigs produced a top BLAST hit to *ORF2*, *Calmodulin*, *XXC finger 6*, *Ribosomal rRNA S20*, *endonuclease reverse transcriptase*, *polyprotein*, and *Tc1-like transposase*. Of these, *Tc1-like transposase *may be of interest as studies on salmon have found similar signatures of selection on this gene [34]. *Tc1-like transposase *is co-regulated with other genes implicated in the immune response, signal transduction, and regulation of transcription. Moreover, *Tc1-like *domains reside in a number of immune and stress-related salmonid genes [34] and probably perform similar functions in lake sturgeon.

#### Rarefaction and normalization

Rarefaction has proven valuable in ecology and population genetics (27–31); in this paper we demonstrate that rarefaction is also useful in the context of gene discovery. In the lake sturgeon, every 5.2 reads (on average) resulted in a different significant BLAST hit (Figure 5). To truly evaluate gene expression, sequencing effort must be sufficient to detect each RNA species and its dosage. In other words, the absolute depth of sequencing coverage required is a function of the total number of transcripts and their relative abundance. cDNA normalization is often employed when gene discovery (as opposed to gene dosage) is the primary consideration [7,10]. Normalization effectively reduces the expression of common genes, thus enhancing detection of rare transcripts relative to a native library. However, in many cases (e.g., sex determining genes) it may not be clear *a priori *if dosage is important [35]. Our data indicate the depth of sequencing coverage provided by next-generation sequencing platforms may obviate the need for normalization. For example, the proportion of singletons following contig assembly was ~65% of the reads in the normalized libraries and ~67% in the native libraries. Thus, normalization has little impact on the discovery of rare transcripts when thousands of sequences are considered. The rarefaction curves in Figure 5b illustrate this point; after ~5000 sequences, there is no real advantage to normalization. Given that next-generation pyrosequencing approaches all generate tens or hundreds of thousands of sequences, our data suggest that normalization is not usually necessary and can obscure the relative gene expression data that can be inferred from native pools of cDNA.

## Conclusion

We have demonstrated that it is possible to use 454 sequencing methods to rapidly characterize transcriptomes in non-model species of conservation concern, notably a polyploid vertebrate with a large genome. We have identified over 5000 ESTs from lake sturgeon that are represented by similar sequences in GenBank; we also identified 877 candidate SNPs. We used Ts/Tv ratios to identify specific ESTs that might have been targets of selection, and we determined that lake sturgeon SNPs occur (on average) every few hundred bp in transcribed genes. Titration runs indicate that normalization is not normally necessary when thousands of sequences from a single tissue type are considered. Finally, we have utilized ecological rarefaction in a genomics context to gauge the relative intensity of sampling required for gene discovery (i.e., sequencing depth). Overall, pyrosequencing has great potential to rapidly identify genes of interest to ecologists and evolutionary biologists, including those from species of conservation concern.

## Methods

### RNA isolation and cDNA library construction

Gonads were sampled from five different lake sturgeon. One sample was collected from a juvenile lake sturgeon of unknown sex estimated to be 3–4 years of age; this fish died in the Purdue hatchery one night and was frozen whole the following morning for subsequent biopsy. Two adolescent males and two adolescent female lake sturgeon were sampled from Lake Oneida, New York in June 2007 and May 2008. Based on stocking records and established length-at-age relationships [33], lake sturgeon collected in 2007 were most likely 12 years of age and those in 2008 were 13. From these four fish, we collected gonad biopsies with the surgical assistance of veterinarians from Cornell University. The two male fish expressed milt during handling and were apparently ready to mate, whereas gametes from the two females were not fully developed [36]. Biopsies were immediately frozen in liquid nitrogen and RNA was extracted using TRIZOL reagent (Invitrogen) following the manufacturer's protocol but with the addition of an extra isolation step: following homogenization, insoluble material was removed by centrifugation at 12,000 × g for 10 minutes. The resulting supernatant containing the RNA was removed and the protocol proceeded as normal. The resulting RNA pellet was resuspended in 50 μl of RNase free water. Quantity and quality of total RNA was analysed using a spectrophotometer (Nanodrop) and by gel electrophoresis.

Five cDNA libraries were constructed. Library 1 was from the 2007 adolescent male, library 2 from the 2007 adolescent female, library 3 from the unknown juvenile, library 4 from the 2008 adolescent male, and library 5 from the 2008 adolescent female. Libraries 1, 2, and 5 were constructed from 1 μg of RNA whereas libraries 3 and 4 were constructed with 1.5 μg of RNA. Libraries 1 and 2 were constructed using the ClonTech SMART cDNA kit, whereas libraries 3, 4 and 5 used a modified version of the kit: the CDS III/3' primer we used was (5'-TAGAGGCCGAGGCGGCCGACATGTTTTGTTTTTTTTTCTTTTTTTTTTVN) to avoid long homopolymer repeats unsuitable for 454 sequencing. All other primers were unmodified from the ClonTech protocol [37]. For all five libraries, cDNA was amplified using PCR Advantage II polymerase (ClonTech) and the following thermal profile: 1 min at 95°C followed by 23 cycles of 95°C for 7 secs, 68°C for 6 minutes. Five microliters of PCR product were analyzed in a 1% agarose gel to determine amplification efficiency. The entire cDNA pools from samples 3, 4 and 5 were digested with 10 units of *SfiI *for a total of two hours at 50°C because cDNA libraries constructed with ClonTech's SMART kit are susceptible to primer concatemerization, and the *SfiI *enzyme digests both the CDS III/3' and the SMART IV primers. All libraries were purified with the QIAquick PCR Purification Kit (QIAGEN) following manufacturer's instruction and concentrated with a conventional ethanol precipitation. The quality and quantity of the cDNA library was evaluated by both spectrophotometry and by gel electrophoresis. Libraries 1 and 2 were normalized using the Evrogen Trimmer-Direct Kit (Evrogen); normalized cDNA was purified using the QIAquick PCR purification Kit. Libraries 3, 4 and 5 were not normalized.

### 454 sequencing and assembly

Approximately 4 μg of amplified cDNA was used for 454 library construction and sequencing following established protocols [1]. Titration runs on the 454 were conducted on all 5 libraries. Bases were called by measuring the luminescence intensity from each well and comparing it to known standards. Upon completion, sequences were screened for primer concatemers, weak signal, and poly A/T tails.

Commercial software provided with Roche 454 sequencers (i.e., Newbler assembler) performs poorly with non-model species that lack a reference genome sequence [3,38]. Thus, sequence assembly was conducted using the default parameters in PCAP, a free program that works well with next-generation sequence datasets [39]. BLASTx was used to search for similar sequences in GenBank. The top hit of each BLASTx search with an e-value ≤ 1 × 10^-03 ^and a bit score > 40 was considered a significant match. BLAST results were then imported into Blast2GO [40], a software package that retrieves GO terms, allowing the function of ESTs to be determined and compared [40,41]. Significant GO was determined with an e-value ≤ 1 × 10^-03 ^and a bit score > 40. Ontology was categorized with respect to Molecular Function, Biological Process, and Cellular Component.

We statistically evaluated normalization efficiency by calculating the exact binomial probability of obtaining the observed number of counts of a particular gene by chance alone. In other words, our null hypothesis was no statistical difference in the expression of genes between the normalized and native libraries. Genes with a p-value less than 0.05 were more abundant in one of the libraries than expected by chance.

### Rarefaction

We generated rarefaction curves [27,28] to compare the rate of discovery of new genes in the normalized versus native libraries as compared to the amount of effort as measured by sequencing depth. The mean number of genes that produced a significant BLAST hit and the mean number of sequences were calculated for the normalized and native libraries. Rarefaction curves were constructed using resampling procedures similar to bootstrapping: all identified genes within a library were recorded along with their frequency. The list was then randomized; the numbers sorted in ascending order, and the first appearance of the new gene was recorded along with its frequency. This was repeated 1,000 times with the program EcoSim700 [42]. Rarefaction analysis was performed on empirical data (*i.e*., actual counts from the sequences) and up to 10,000 simulated sequences.

### SNP discovery

To identify SNPs, we pooled all sequences from the five libraries into PCAP and constructed contigs from the entire data set. The consensus sequences from these contigs were used as reference sequences to which individual reads were aligned using GC Reference Mapper (454 Life Science). Each read was only aligned to one site in the reference sequence; if reads were aligned to different references sequences then the reads were discarded. We limited SNP scoring to contigs composed of four or more reads, and we only identified SNPs where 30 bp of high-quality sequence data was present both upstream and downstream of the variable site. We also discounted SNPs in homopolymer repeats of > 4 nucleotides. The Ts/Tv ratios were determined for every SNP-containing contig that BLASTed to a known gene.

## Authors' contributions

JAD conceived and designed the research plan. JRJ led the field expeditions and coordinated the gonad sampling. MCH and CRM carried out lab work and constructed the cDNA libraries. MCH analysed and interpreted the sequence data. MCH and JAD drafted the manuscript. All authors contributed to the content of the manuscript, and have read and approved the final version.

## Supplementary Material

Additional File 1 **Differences in expression of genes between normalized and native libraries (Molecular Function categories)**. List of genes found within the Molecular function category of the Gene Ontology assignment with a significant difference in expression between normalized and native libraries. Click here for file

Additional file 2 **Differences in expression of genes between normalized and native libraries (Biological Process categories)**. List of genes found within the Biological Process category of the Gene Ontology assignment. Click here for file

Additional file 3 **Differences in expression of genes between normalized and native libraries (Cellular Component categories)**. List of genes found within the Cellular Component category of the Gene Ontology assignment. Click here for file

Additional file 4 **Details of all SNPs detected in contigs**. List of contigs with associated contig length, contig depth, number of SNPs and Ts/Tv ratio. Click here for file

Additonal File 5 **Details of all SNPs in contigs with a signficant BLAST hit**. Subset of contigs with a significant BLAST hit **and **at least one SNP. In other words, the SNPs described in this table are a subset of the 877 we identified, but include all of those associated with a particular gene. Click here for file
