# Supplementary material for: Next-generation pyrosequencing of gonad transcriptomes in the polyploid lake sturgeon (Acipenser fulvescens): the relative merits of normalization and rarefaction in gene discovery
Source: BMC Genomics. 2009 Apr 29;10:203. doi: 10.1186/1471-2164-10-203 (PMC2688523; doi:10.1186/1471-2164-10-203)
Supplement: Additional File 1 — Differences in expression of genes between normalized and native libraries (Molecular Function categories). List of genes found within the Molecular function category of the Gene Ontology assignment with a significant difference in expression between normalized and native libraries. [file 1471-2164-10-203-S1.doc]

| **Molecular Function** | **Total counts normalized** | **% counts** | **Total counts native** | **% counts** | **P-value normalized libraries** | **P-value native libraries** |
| --- | --- | --- | --- | --- | --- | --- |
| 5S RNA binding | 0 | 0.00 | 157 | 3.48 |  | 0.084 |
| actin binding | 7 | 1.39 | 24 | 0.53 | 0.014 |  |
| actin cytoskeleton | 0 | 0.00 | 6 | 0.13 |  | 0.298 |
| acyl-CoA activity | 0 | 0.00 | 4 | 0.09 |  | 0.293 |
| acyltransferase activity | 1 | 0.20 | 4 | 0.09 | 0.090 |  |
| anatomical structure morphogenesis | 0 | 0.00 | 5 | 0.11 |  | 0.297 |
| androgen receptor signaling pathway | 0 | 0.00 | 6 | 0.13 |  | 0.298 |
| anterograde axon cargo transport | 0 | 0.00 | 2 | 0.04 | 0.182 |  |
| ARF | 0 | 0.00 | 3 | 0.07 | 0.260 |  |
| arginine | 0 | 0.00 | 6 | 0.13 |  | 0.298 |
| ATP binding | 101 | 20.12 | 152 | 3.37 | 0.001 |  |
| ATP synthesis | 0 | 0.00 | 10 | 0.22 |  | 0.294 |
| ATPase activity | 0 | 0.00 | 12 | 0.27 |  | 0.289 |
| ATP-dependent proteolysis | 0 | 0.00 | 4 | 0.09 |  | 0.293 |
| axongenesis | 0 | 0.00 | 9 | 0.20 |  | 0.296 |
| barbed-end actin filament capping | 0 | 0.00 | 11 | 0.24 |  | 0.292 |
| binding | 0 | 0.00 | 29 | 0.64 |  | 0.246 |
| biopolymer metabolic process | 0 | 0.00 | 3 | 0.07 | 0.260 |  |
| blood vessel endothelial cell migration | 0 | 0.00 | 6 | 0.13 |  | 0.298 |
| brush border | 0 | 0.00 | 4 | 0.09 |  | 0.293 |
| calcium channel activity | 0 | 0.00 | 7 | 0.16 |  | 0.298 |
| calmodium binding | 0 | 0.00 | 9 | 0.20 |  | 0.296 |
| carbohydrate binding | 0 | 0.00 | 4 | 0.09 |  | 0.293 |
| caspase activation | 0 | 0.00 | 4 | 0.09 |  | 0.293 |
| catalytic activity | 1 | 0.20 | 4 | 0.09 | 0.090 |  |
| cathepsin B activity | 0 | 0.00 | 2 | 0.04 | 0.182 |  |
| chromatin binding | 2 | 0.40 | 20 | 0.44 | 0.379 |  |
| clathrin | 0 | 0.00 | 11 | 0.24 |  | 0.292 |
| coated pit | 0 | 0.00 | 6 | 0.13 |  | 0.298 |
| coenzyme binding | 0 | 0.00 | 6 | 0.13 |  | 0.298 |
| copper ion binding | 0 | 0.00 | 140 | 3.11 |  | 0.096 |
| cyt-c oxidase | 78 | 15.54 | 155 | 3.44 | 0.001 |  |
| cytokine activity | 3 | 0.60 | 13 | 0.29 | 0.079 |  |
| diacylglycerol binding | 0 | 0.00 | 3 | 0.07 | 0.260 |  |
| DNA binding | 23 | 4.58 | 76 | 1.69 | 0.001 |  |
| double-strand DNA binding | 0 | 0.00 | 7 | 0.16 |  | 0.298 |
| endocytosis | 0 | 0.00 | 180 | 3.99 |  | 0.071 |
| exonuclease activity | 0 | 0.00 | 5 | 0.11 |  | 0.297 |
| fatty-acid binding | 0 | 0.00 | 15 | 0.33 |  | 0.282 |
| ferric iron binding | 0 | 0.00 | 4 | 0.09 |  | 0.293 |
| ferroxidase activity | 0 | 0.00 | 4 | 0.09 |  | 0.293 |
| Glutathione process | 0 | 0.00 | 16 | 0.35 |  | 0.279 |
| glutamate binding | 0 | 0.00 | 22 | 0.49 |  | 0.263 |
| GTP binding | 6 | 1.20 | 22 | 0.49 | 0.024 |  |
| GTPase activator | 0 | 0.00 | 23 | 0.51 |  | 0.261 |
| heat shock protein | 0 | 0.00 | 4 | 0.09 |  | 0.293 |
| heparin binding | 0 | 0.00 | 10 | 0.22 |  | 0.294 |
| histone acetylation | 0 | 0.00 | 6 | 0.13 |  | 0.298 |
| histone methylation | 0 | 0.00 | 6 | 0.13 |  | 0.298 |
| hydrogen ion transporting ATP | 0 | 0.00 | 10 | 0.22 |  | 0.294 |
| icosatetraenoic acid binding | 0 | 0.00 | 5 | 0.11 |  | 0.297 |
| I-kappaB kinase/NF-kappaB cascade | 0 | 0.00 | 49 | 1.09 |  | 0.204 |
| induction of apoptosis | 0 | 0.00 | 12 | 0.27 |  | 0.289 |
| insulin binding factor | 0 | 0.00 | 6 | 0.13 |  | 0.298 |
| iron ion binding | 0 | 0.00 | 45 | 1.00 |  | 0.212 |
| iron ion transport | 0 | 0.00 | 9 | 0.20 |  | 0.296 |
| isomerase activity | 4 | 0.80 | 8 | 0.18 | 0.008 |  |
| kinase activity | 4 | 0.80 | 5 | 0.11 | 0.002 |  |
| ligase activity | 0 | 0.00 | 4 | 0.09 |  | 0.293 |
| lipid binding | 1 | 0.20 | 7 | 0.16 | 0.192 |  |
| magnesium ion binding | 0 | 0.00 | 18 | 0.40 |  | 0.274 |
| malate metabolic process | 0 | 0.00 | 2 | 0.04 | 0.182 |  |
| manganese ion binding | 0 | 0.00 | 5 | 0.11 |  | 0.297 |
| MAPK activity | 0 | 0.00 | 3 | 0.07 | 0.260 |  |
| metal ion binding | 0 | 0.00 | 16 | 0.35 |  | 0.279 |
| motor activity | 4 | 0.80 | 0 | 0.00 | 0.001 |  |
| mRNA processing | 0 | 0.00 | 16 | 0.35 |  | 0.279 |
| myofibril assembly | 0 | 0.00 | 3 | 0.07 | 0.260 |  |
| negative regulation of apoptosis | 0 | 0.00 | 14 | 0.31 |  | 0.284 |
| negative regulation of caspase activity | 0 | 0.00 | 16 | 0.35 |  | 0.279 |
| negative regulation of cell proliferation | 0 | 0.00 | 20 | 0.44 |  | 0.269 |
| negative regulation to antigenic stimulus | 0 | 0.00 | 4 | 0.09 |  | 0.293 |
| tyrosine kinase activity | 0 | 0.00 | 7 | 0.16 |  | 0.298 |
| oxidoreductase activity | 0 | 0.00 | 16 | 0.35 |  | 0.279 |
| oxygen binding | 12 | 2.39 | 2 | 0.04 | 0.001 |  |
| PDZ domain binding | 0 | 0.00 | 4 | 0.09 |  | 0.293 |
| peptidase activity | 0 | 0.00 | 6 | 0.13 |  | 0.298 |
| peptide binding | 0 | 0.00 | 14 | 0.31 |  | 0.284 |
| phosphate binding | 2 | 0.40 | 9 | 0.20 | 0.100 |  |
| phosphatidylinositol binding | 0 | 0.00 | 10 | 0.22 |  | 0.294 |
| phospholipase inhibitor activity | 0 | 0.00 | 4 | 0.09 |  | 0.293 |
| phospholipid binding | 0 | 0.00 | 5 | 0.11 |  | 0.297 |
| protein amino acid glycosylation | 0 | 0.00 | 4 | 0.09 |  | 0.293 |
| protein amino acid oxidation | 0 | 0.00 | 13 | 0.29 |  | 0.287 |
| protein amino acid phosphorylation | 0 | 0.00 | 25 | 0.55 |  | 0.256 |
| protein binding | 178 | 35.46 | 1348 | 29.90 | 0.007 |  |
| protein heterodimerization activity | 0 | 0.00 | 10 | 0.22 |  | 0.294 |
| protein kinase c | 0 | 0.00 | 14 | 0.31 |  | 0.284 |
| protein phosphatase regulator activity | 0 | 0.00 | 5 | 0.11 |  | 0.297 |
| protein serine/threonine kinase activity | 0 | 0.00 | 16 | 0.35 |  | 0.279 |
| protein tyrosine phosphatase activator activity | 0 | 0.00 | 4 | 0.09 |  | 0.293 |
| proteinaceous extracellular matrix | 0 | 0.00 | 6 | 0.13 |  | 0.298 |
| purine base biosynthetic process | 0 | 0.00 | 10 | 0.22 |  | 0.294 |
| pyridoxal phosphate binding | 0 | 0.00 | 7 | 0.16 |  | 0.298 |
| receptor activity | 0 | 0.00 | 33 | 0.73 |  | 0.237 |
| release of cyt c from mitochondria | 0 | 0.00 | 4 | 0.09 |  | 0.293 |
| rhodopsin-like receptor activity | 0 | 0.00 | 2 | 0.04 | 0.182 |  |
| ribosomal protein import into nucleus | 0 | 0.00 | 4 | 0.09 |  | 0.293 |
| ribosome biogenesis | 0 | 0.00 | 811 | 17.99 |  | 0.001 |
| RNA binding | 33 | 6.57 | 1482 | 32.87 |  | 0.001 |
| selenium binding | 0 | 0.00 | 19 | 0.42 |  | 0.271 |
| signal peptidase activity | 0 | 0.00 | 8 | 0.18 |  | 0.297 |
| signal transducer activity | 0 | 0.00 | 11 | 0.24 |  | 0.292 |
| small GTPase mediated signal transduction | 0 | 0.00 | 10 | 0.22 |  | 0.294 |
| structural constituent of cytoskeleton | 0 | 0.00 | 8 | 0.18 |  | 0.297 |
| structural constituent of ribosome | 0 | 0.00 | 1791 | 39.73 |  | 0.001 |
| sugar binding | 2 | 0.40 | 6 | 0.13 | 0.048 |  |
| transcription regulator activity | 0 | 0.00 | 16 | 0.35 |  | 0.279 |
| transposase activity | 0 | 0.00 | 38 | 0.84 |  | 0.226 |
| ubiquinol-cytochrome-c reductase activity | 22 | 4.38 | 6 | 0.13 | 0.001 |  |
| unfolded protein binding | 0 | 0.00 | 20 | 0.44 |  | 0.269 |
| UV protection | 0 | 0.00 | 16 | 0.35 |  | 0.279 |
| zinc ion binding | 136 | 27.09 | 725 | 16.08 | 0.001 |  |
