# Supplementary material for: Next-generation pyrosequencing of gonad transcriptomes in the polyploid lake sturgeon (Acipenser fulvescens): the relative merits of normalization and rarefaction in gene discovery
Source: BMC Genomics. 2009 Apr 29;10:203. doi: 10.1186/1471-2164-10-203 (PMC2688523; doi:10.1186/1471-2164-10-203)
Supplement: Additional file 2 — Differences in expression of genes between normalized and native libraries (Biological Process categories). List of genes found within the Biological Process category of the Gene Ontology assignment. [file 1471-2164-10-203-S2.doc]

| **Biological Process** | **Total counts normalized libraries** | **% counts** | **Total counts native libraries** | **% counts** | **P-value normalized libraries** | **P-value native libraries** |
| --- | --- | --- | --- | --- | --- | --- |
| aerobic respiration | 0 | 0.00 | 37 | 0.82 |  | 0.313 |
| angiogenesis | 0 | 0.00 | 20 | 0.44 |  | 0.335 |
| anti-apoptosis | 0 | 0.00 | 9 | 0.20 |  | 0.341 |
| apoptosis | 6 | 2.08 | 8 | 0.18 | 0.001 |  |
| biosynthetic process | 14 | 4.84 | 2 | 0.04 | 0.001 |  |
| calcium ion | 0 | 0.00 | 62 | 1.38 |  | 0.283 |
| cell adhesion | 8 | 2.77 | 11 | 0.24 | 0.001 |  |
| cell aging | 0 | 0.00 | 7 | 0.16 |  | 0.339 |
| cell cortex | 0 | 0.00 | 4 | 0.09 | 0.215 |  |
| cell division | 0 | 0.00 | 7 | 0.16 |  | 0.339 |
| cell projection | 0 | 0.00 | 16 | 0.36 |  | 0.339 |
| cell proliferation | 0 | 0.00 | 20 | 0.44 |  | 0.335 |
| cell ion homeostasis | 0 | 0.00 | 14 | 0.31 |  | 0.341 |
| chromatin modification | 0 | 0.00 | 12 | 0.27 |  | 0.342 |
| biogenesis | 0 | 0.00 | 174 | 3.87 |  | 0.190 |
| DNA integration | 0 | 0.00 | 17 | 0.38 |  | 0.338 |
| DNA repair | 14 | 4.84 | 9 | 0.20 | 0.001 |  |
| dopamine metabolism | 0 | 0.00 | 6 | 0.13 | 0.304 |  |
| dosage compensation | 0 | 0.00 | 2 | 0.04 | 0.114 |  |
| electron transport | 7 | 2.42 | 203 | 4.51 |  | 0.323 |
| endothelial cell development | 0 | 0.00 | 15 | 0.33 |  | 0.340 |
| glycoloysis | 7 | 2.42 | 8 | 0.18 | 0.001 |  |
| heart contraction | 0 | 0.00 | 7 | 0.16 |  | 0.339 |
| heme binding | 0 | 0.00 | 136 | 3.02 |  | 0.216 |
| hydrogen peroxide catabolic process | 0 | 0.00 | 17 | 0.38 |  | 0.338 |
| immune response | 7 | 2.42 | 6 | 0.13 | 0.001 |  |
| intracellular protein transport | 0 | 0.00 | 15 | 0.33 |  | 0.340 |
| intracellular protein membrane transport | 0 | 0.00 | 8 | 0.18 |  | 0.340 |
| long-chain fatty acid biosynthetic process | 0 | 0.00 | 16 | 0.36 |  | 0.339 |
| macromolecule complex | 0 | 0.00 | 4 | 0.09 | 0.215 |  |
| metabolic process | 26 | 9.00 | 4 | 0.09 | 0.001 |  |
| mitochondrial electron transport | 0 | 0.00 | 23 | 0.51 |  | 0.331 |
| mitochondrial respiratory chain | 0 | 0.00 | 180 | 4.00 |  | 0.186 |
| multicellular organismal development | 0 | 0.00 | 20 | 0.44 |  | 0.335 |
| muscle cell differentiation | 95 | 32.87 | 4 | 0.09 | 0.001 |  |
| NADH ubiquinone activity | 0 | 0.00 | 30 | 0.67 |  | 0.322 |
| Notch signaling pathway | 0 | 0.00 | 6 | 0.13 | 0.304 |  |
| nucleic acid binding | 0 | 0.00 | 20 | 0.44 |  | 0.335 |
| nucleotide binding | 0 | 0.00 | 28 | 0.62 |  | 0.325 |
| positive regulation of apoptosis | 3 | 1.04 | 6 | 0.13 | 0.002 |  |
| positive regulation of transcription | 0 | 0.00 | 25 | 0.56 |  | 0.329 |
| protein complex | 0 | 0.00 | 25 | 0.56 |  | 0.329 |
| protein folding | 15 | 5.19 | 25 | 0.56 | 0.001 |  |
| protein transport | 4 | 1.38 | 17 | 0.38 | 0.009 |  |
| proteolysis | 6 | 2.08 | 21 | 0.47 | 0.001 |  |
| proton transport | 0 | 0.00 | 14 | 0.31 |  | 0.341 |
| regulation of cell redox homeostasis | 0 | 0.00 | 16 | 0.36 |  | 0.339 |
| regulation of cell shape | 0 | 0.00 | 610 | 13.55 |  | 0.049 |
| regulation of epithelial cell proliferation | 0 | 0.00 | 16 | 0.36 |  | 0.339 |
| regulation of transcription | 0 | 0.00 | 70 | 1.56 |  | 0.274 |
| regulation of translation | 0 | 0.00 | 15 | 0.33 |  | 0.340 |
| response to oxidative stress | 0 | 0.00 | 13 | 0.29 |  | 0.341 |
| response to selenium ion | 0 | 0.00 | 15 | 0.33 |  | 0.340 |
| response to stress | 0 | 0.00 | 26 | 0.58 |  | 0.327 |
| sensory perception of sound | 0 | 0.00 | 25 | 0.56 |  | 0.329 |
| skeletal muscle regeneration | 0 | 0.00 | 15 | 0.33 |  | 0.340 |
| spermatogenesis | 0 | 0.00 | 20 | 0.44 |  | 0.335 |
| T cell activity | 0 | 0.00 | 52 | 1.16 |  | 0.295 |
| transcription | 0 | 0.00 | 116 | 2.58 |  | 0.231 |
| translation | 37 | 12.80 | 1830 | 40.66 |  | 0.010 |
| transport | 40 | 13.84 | 211 | 4.69 | 0.001 |  |
| transposition | 0 | 0.00 | 41 | 0.91 |  | 0.308 |
| ubiquitin | 0 | 0.00 | 63 | 1.40 |  | 0.282 |
| vasculogenesis | 0 | 0.00 | 9 | 0.20 |  | 0.341 |
| vesicle-mediated transport | 0 | 0.00 | 8 | 0.18 |  | 0.340 |
| zinc ion transport | 0 | 0.00 | 10 | 0.22 |  | 0.342 |
