# Supplementary material for: Next-generation pyrosequencing of gonad transcriptomes in the polyploid lake sturgeon (Acipenser fulvescens): the relative merits of normalization and rarefaction in gene discovery
Source: BMC Genomics. 2009 Apr 29;10:203. doi: 10.1186/1471-2164-10-203 (PMC2688523; doi:10.1186/1471-2164-10-203)
Supplement: Additional file 3 — Differences in expression of genes between normalized and native libraries (Cellular Component categories). List of genes found within the Cellular Component category of the Gene Ontology assignment. [file 1471-2164-10-203-S3.doc]

| **Cellular Component** | **Total counts normalized libraries** | **% counts** | **Total counts native libraries** | **% counts** | **P-value normalized** | **P-value native** |
| --- | --- | --- | --- | --- | --- | --- |
| actin filament | 93 | 12.77 | 24 | 0.65 | 0.00 |  |
| apical plasma membrane | 0 | 0.00 | 7 | 0.19 |  | 0.23 |
| barr body | 0 | 0.00 | 2 | 0.05 |  | 0.23 |
| basal plasma membrane | 0 | 0.00 | 1 | 0.03 | 0.15 |  |
| basement membrane | 0 | 0.00 | 4 | 0.11 |  | 0.24 |
| centrosome | 0 | 0.00 | 5 | 0.14 |  | 0.24 |
| chromosome | 0 | 0.00 | 2 | 0.05 |  | 0.23 |
| collagen | 4 | 0.55 | 13 | 0.35 | 0.15 |  |
| cytoplasm | 187 | 25.69 | 225 | 6.10 | 0.00 |  |
| cytoskeleton | 0 | 0.00 | 33 | 0.90 |  | 0.13 |
| cytosol | 46 | 6.32 | 42 | 1.14 | 0.00 |  |
| cytosolic large ribosomal subunit | 0 | 0.00 | 193 | 5.23 |  | 0.01 |
| cytosolic ribosome | 0 | 0.00 | 75 | 2.03 |  | 0.05 |
| cytosolic small subunit | 0 | 0.00 | 810 | 21.97 |  | 0.00 |
| dendrite | 0 | 0.00 | 8 | 0.22 |  | 0.23 |
| ER | 16 | 2.20 | 43 | 1.17 | 0.02 |  |
| endosome | 0 | 0.00 | 7 | 0.19 |  | 0.23 |
| extracellular region | 20 | 2.75 | 90 | 2.44 | 0.28 |  |
| golgi apparatus | 9 | 1.24 | 20 | 0.54 | 0.02 |  |
| golgi lumen | 0 | 0.00 | 1 | 0.03 | 0.15 |  |
| golgi membrane | 0 | 0.00 | 8 | 0.22 |  | 0.23 |
| golgi stack | 0 | 0.00 | 7 | 0.19 |  | 0.23 |
| G-protein receptor | 0 | 0.00 | 8 | 0.22 |  | 0.23 |
| golgi membrane | 0 | 0.00 | 5 | 0.14 |  | 0.24 |
| integral to membrane | 47 | 6.46 | 233 | 6.32 | 0.41 |  |
| Integral plasma membrane | 0 | 0.00 | 15 | 0.41 |  | 0.20 |
| intracellular | 0 | 0.00 | 23 | 0.62 |  | 0.16 |
| intracellular membrane | 0 | 0.00 | 8 | 0.22 |  | 0.23 |
| large ribosomal subunit | 0 | 0.00 | 12 | 0.33 |  | 0.21 |
| lipid particle | 0 | 0.00 | 7 | 0.19 |  | 0.23 |
| lysosome | 3 | 0.41 | 8 | 0.22 | 0.11 |  |
| melanosome | 0 | 0.00 | 23 | 0.62 |  | 0.16 |
| membrane | 110 | 15.11 | 239 | 6.48 | 0.00 |  |
| microsome | 0 | 0.00 | 17 | 0.46 |  | 0.19 |
| microtubule | 0 | 0.00 | 22 | 0.60 |  | 0.17 |
| microtubule cytoskeleton | 0 | 0.00 | 3 | 0.08 |  | 0.24 |
| microvilus | 0 | 0.00 | 3 | 0.08 |  | 0.24 |
| mitochondrial inner membrane | 0 | 0.00 | 27 | 0.73 |  | 0.15 |
| mitochondrial large ribosomal subunit | 0 | 0.00 | 3 | 0.08 |  | 0.24 |
| mitochondrial matrix | 0 | 0.00 | 11 | 0.30 |  | 0.22 |
| mitochondrion | 111 | 15.25 | 66 | 1.79 | 0.00 |  |
| holoenzyme complex | 0 | 0.00 | 2 | 0.05 |  | 0.23 |
| nuclear matrix | 0 | 0.00 | 6 | 0.16 |  | 0.24 |
| nuclear membrane | 0 | 0.00 | 2 | 0.05 |  | 0.23 |
| nuclear pore | 0 | 0.00 | 6 | 0.16 |  | 0.24 |
| nuclear speck | 0 | 0.00 | 9 | 0.24 |  | 0.23 |
| nucleolus | 0 | 0.00 | 32 | 0.87 |  | 0.13 |
| nucleoplasm | 0 | 0.00 | 8 | 0.22 |  | 0.23 |
| nucleosome | 0 | 0.00 | 17 | 0.46 |  | 0.19 |
| nucleus | 38 | 5.22 | 241 | 6.54 |  | 0.28 |
| perinuclear region of cytoplasm | 15 | 2.06 | 19 | 0.52 | 0.00 |  |
| peroxisome | 0 | 0.00 | 15 | 0.41 |  | 0.20 |
| plasma membrane | 4 | 0.55 | 207 | 5.61 |  | 0.01 |
| pronucleus | 0 | 0.00 | 3 | 0.08 |  | 0.24 |
| proteasome | 0 | 0.00 | 20 | 0.54 |  | 0.17 |
| ribonucleoprotein complex | 0 | 0.00 | 11 | 0.30 |  | 0.22 |
| ribosome | 25 | 3.43 | 645 | 17.49 |  | 0.00 |
| small ribosomal subunit | 0 | 0.00 | 64 | 1.74 |  | 0.07 |
| spindle pole | 0 | 0.00 | 2 | 0.05 |  | 0.23 |
| stress fiber | 0 | 0.00 | 2 | 0.05 |  | 0.23 |
| striated muscle thin filament | 0 | 0.00 | 2 | 0.05 |  | 0.23 |
| synapse | 0 | 0.00 | 21 | 0.57 |  | 0.17 |
