# Supplementary material for: Next-generation pyrosequencing of gonad transcriptomes in the polyploid lake sturgeon (Acipenser fulvescens): the relative merits of normalization and rarefaction in gene discovery
Source: BMC Genomics. 2009 Apr 29;10:203. doi: 10.1186/1471-2164-10-203 (PMC2688523; doi:10.1186/1471-2164-10-203)
Supplement: Additional file 4 — Details of all SNPs detected in contigs. List of contigs with associated contig length, contig depth, number of SNPs and Ts/Tv ratio. [file 1471-2164-10-203-S4.doc]

| **Contig name** | **Contig length** | **Contig depth** | **Number of SNPs** | **SNPs/bp** | **Ts** | **Tv** | **Ts/Tv ratio** |
| --- | --- | --- | --- | --- | --- | --- | --- |
| 0.1 | 1823 | 175 | 38 | 48.0 | 24 | 10 | 2.4 |
| 1.1 | 1201 | 142 | 45 | 26.7 | 28 | 16 | 1.8 |
| 2.1 | 652 | 85 | 41 | 15.9 | 30 | 11 | 2.7 |
| 3.1 | 527 | 68 | 11 | 47.9 | 7 | 4 | 1.8 |
| 4.1 | 272 | 57 | 16 | 17.0 | 8 | 8 | 1.0 |
| 5.1 | 730 | 56 | 31 | 23.5 | 24 | 7 | 3.4 |
| 6.1 | 329 | 48 | 5 | 65.8 | 5 | 0 | 5.0 |
| 8.1 | 704 | 43 | 14 | 50.3 | 9 | 5 | 1.8 |
| 9.1 | 650 | 43 | 25 | 26.0 | 16 | 8 | 2.0 |
| 10.1 | 538 | 43 | 6 | 89.7 | 5 | 1 | 5.0 |
| 11.1 | 923 | 41 | 20 | 46.2 | 15 | 4 | 3.8 |
| 12.1 | 1072 | 40 | 10 | 107.2 | 9 | 1 | 9.0 |
| 14.1 | 819 | 29 | 6 | 136.5 | 4 | 2 | 2.0 |
| 15.1 | 522 | 29 | 15 | 34.8 | 9 | 6 | 1.5 |
| 16.1 | 311 | 29 | 7 | 44.4 | 5 | 2 | 2.5 |
| 17.1 | 400 | 28 | 4 | 100.0 | 4 | 0 | 4.0 |
| 18.1 | 421 | 28 | 5 | 84.2 | 2 | 3 | 0.7 |
| 19.1 | 379 | 27 | 8 | 47.4 | 1 | 2 | 0.5 |
| 20.1 | 581 | 27 | 8 | 72.6 | 5 | 3 | 1.7 |
| 21.1 | 315 | 27 | 11 | 28.6 | 7 | 4 | 1.8 |
| 22.1 | 751 | 26 | 7 | 107.3 | 5 | 2 | 2.5 |
| 24.1 | 446 | 26 | 10 | 44.6 | 5 | 5 | 1.0 |
| 25.1 | 886 | 24 | 1 | 886.0 | 1 | 0 | 1.0 |
| 26.1 | 630 | 24 | 2 | 315.0 | 0 | 2 | 0.0 |
| 27.1 | 619 | 24 | 17 | 36.4 | 9 | 7 | 1.3 |
| 28.1 | 656 | 24 | 9 | 72.9 | 5 | 4 | 1.3 |
| 29.1 | 321 | 23 | 15 | 21.4 | 2 | 13 | 0.2 |
| 32.1 | 411 | 22 | 7 | 58.7 | 5 | 2 | 2.5 |
| 35.1 | 325 | 20 | 1 | 325.0 | 1 | 0 | 1.0 |
| 36.1 | 691 | 20 | 2 | 345.5 | 2 | 0 | 2.0 |
| 37.1 | 433 | 19 | 4 | 108.3 | 3 | 1 | 3.0 |
| 38.1 | 692 | 19 | 7 | 98.9 | 3 | 4 | 0.8 |
| 39.1 | 599 | 19 | 3 | 199.7 | 2 | 1 | 2.0 |
| 40.1 | 738 | 19 | 11 | 67.1 | 6 | 5 | 1.2 |
| 42.1 | 567 | 18 | 21 | 27.0 | 11 | 9 | 1.2 |
| 43.1 | 501 | 17 | 5 | 100.2 | 2 | 2 | 1.0 |
| 44.1 | 413 | 17 | 10 | 41.3 | 9 | 1 | 9.0 |
| 45.1 | 322 | 17 | 5 | 64.4 | 2 | 3 | 0.7 |
| 46.1 | 619 | 17 | 9 | 68.8 | 8 | 1 | 8.0 |
| 47.1 | 256 | 17 | 10 | 25.6 | 6 | 4 | 1.5 |
| 48.1 | 343 | 16 | 3 | 114.3 | 3 | 0 | 3.0 |
| 50.1 | 526 | 16 | 6 | 87.7 | 5 | 1 | 5.0 |
| 51.1 | 408 | 16 | 2 | 204.0 | 1 | 1 | 1.0 |
| 52.1 | 466 | 15 | 3 | 155.3 | 1 | 2 | 0.5 |
| 55.1 | 463 | 15 | 5 | 92.6 | 4 | 1 | 4.0 |
| 58.1 | 415 | 14 | 5 | 83.0 | 3 | 2 | 1.5 |
| 60.1 | 293 | 14 | 3 | 97.7 | 2 | 1 | 2.0 |
| 61.1 | 465 | 13 | 1 | 465.0 | 0 | 1 | 0.0 |
| 63.1 | 345 | 13 | 4 | 86.3 | 3 | 1 | 3.0 |
| 65.1 | 553 | 13 | 7 | 79.0 | 3 | 4 | 0.8 |
| 66.1 | 362 | 13 | 3 | 120.7 | 3 | 0 | 3.0 |
| 67.1 | 565 | 13 | 6 | 94.2 | 4 | 2 | 2.0 |
| 76.1 | 399 | 12 | 1 | 399.0 | 1 | 0 | 1.0 |
| 80.1 | 314 | 12 | 1 | 314.0 | 1 | 0 | 1.0 |
| 83.1 | 219 | 12 | 8 | 27.4 | 7 | 1 | 7.0 |
| 88.1 | 539 | 11 | 1 | 539.0 | 1 | 0 | 1.0 |
| 89.1 | 289 | 11 | 3 | 96.3 | 3 | 0 | 3.0 |
| 92.1 | 606 | 11 | 9 | 67.3 | 7 | 2 | 3.5 |
| 95.1 | 178 | 11 | 2 | 89.0 | 2 | 0 | 2.0 |
| 97.1 | 470 | 11 | 7 | 67.1 | 4 | 3 | 1.3 |
| 98.1 | 310 | 10 | 12 | 25.8 | 9 | 3 | 3.0 |
| 100.1 | 404 | 10 | 3 | 134.7 | 3 | 0 | 3.0 |
| 101.1 | 327 | 10 | 12 | 27.3 | 10 | 2 | 5.0 |
| 102.1 | 364 | 10 | 7 | 52.0 | 4 | 3 | 1.3 |
| 113.1 | 469 | 10 | 2 | 234.5 | 1 | 1 | 1.0 |
| 116.1 | 421 | 9 | 1 | 421.0 | 0 | 1 | 0.0 |
| 124.1 | 285 | 9 | 1 | 285.0 | 1 | 0 | 1.0 |
| 127.1 | 296 | 9 | 12 | 24.7 | 9 | 3 | 3.0 |
| 130.1 | 521 | 9 | 8 | 65.1 | 4 | 4 | 1.0 |
| 134.1 | 285 | 8 | 3 | 95.0 | 2 | 1 | 2.0 |
| 137.1 | 501 | 8 | 2 | 250.5 | 1 | 1 | 1.0 |
| 138.1 | 412 | 8 | 4 | 103.0 | 3 | 1 | 3.0 |
| 140.1 | 263 | 8 | 3 | 87.7 | 2 | 1 | 2.0 |
| 143.1 | 312 | 8 | 6 | 52.0 | 1 | 5 | 0.2 |
| 144.1 | 197 | 8 | 2 | 98.5 | 2 | 0 | 2.0 |
| 145.1 | 423 | 8 | 1 | 423.0 | 0 | 1 | 0.0 |
| 146.1 | 274 | 8 | 2 | 137.0 | 1 | 1 | 1.0 |
| 147.1 | 446 | 8 | 6 | 74.3 | 2 | 4 | 0.5 |
| 148.1 | 120 | 8 | 3 | 40.0 | 2 | 1 | 2.0 |
| 152.1 | 210 | 8 | 7 | 30.0 | 4 | 3 | 1.3 |
| 153.1 | 437 | 8 | 7 | 62.4 | 6 | 1 | 6.0 |
| 156.1 | 365 | 8 | 11 | 33.2 | 7 | 4 | 1.8 |
| 166.1 | 265 | 7 | 4 | 66.3 | 2 | 2 | 1.0 |
| 168.1 | 519 | 7 | 3 | 173.0 | 2 | 1 | 2.0 |
| 171.1 | 311 | 7 | 2 | 155.5 | 2 | 0 | 2.0 |
| 173.1 | 479 | 7 | 8 | 59.9 | 3 | 5 | 0.6 |
| 176.1 | 311 | 7 | 14 | 22.2 | 4 | 10 | 0.4 |
| 180.1 | 258 | 7 | 2 | 129.0 | 1 | 1 | 1.0 |
| 183.1 | 299 | 7 | 5 | 59.8 | 3 | 2 | 1.5 |
| 186.1 | 433 | 7 | 7 | 61.9 | 5 | 2 | 2.5 |
| 187.1 | 265 | 7 | 4 | 66.3 | 2 | 2 | 1.0 |
| 191.1 | 353 | 7 | 2 | 176.5 | 2 | 0 | 2.0 |
| 192.1 | 480 | 7 | 1 | 480.0 | 1 | 0 | 1.0 |
| 193.1 | 342 | 7 | 5 | 68.4 | 5 | 0 | 5.0 |
| 195.1 | 308 | 7 | 3 | 102.7 | 2 | 1 | 2.0 |
| 215.1 | 467 | 6 | 2 | 233.5 | 1 | 1 | 1.0 |
| 221.1 | 260 | 6 | 4 | 65.0 | 3 | 1 | 3.0 |
| 226.1 | 399 | 6 | 4 | 99.8 | 2 | 2 | 1.0 |
| 228.1 | 455 | 6 | 1 | 455.0 | 0 | 1 | 0.0 |
| 229.1 | 472 | 6 | 7 | 67.4 | 1 | 4 | 0.3 |
| 237.1 | 294 | 6 | 12 | 24.5 | 10 | 2 | 5.0 |
| 239.1 | 250 | 6 | 2 | 125.0 | 2 | 0 | 2.0 |
| 242.1 | 253 | 6 | 3 | 84.3 | 1 | 1 | 1.0 |
| 244.1 | 224 | 6 | 1 | 224.0 | 1 | 0 | 1.0 |
| 251.1 | 247 | 6 | 7 | 35.3 | 3 | 3 | 1.0 |
| 258.1 | 353 | 5 | 3 | 117.7 | 3 | 0 | 3.0 |
| 275.1 | 199 | 5 | 1 | 199.0 | 1 | 0 | 1.0 |
| 278.1 | 485 | 5 | 3 | 161.7 | 0 | 2 | 0.0 |
| 279.1 | 323 | 5 | 1 | 323.0 | 1 | 0 | 1.0 |
| 294.1 | 355 | 5 | 3 | 118.3 | 0 | 3 | 0.0 |
| 302.1 | 283 | 5 | 1 | 283.0 | 0 | 1 | 0.0 |
| 314.1 | 197 | 5 | 1 | 197.0 | 0 | 1 | 0.0 |
| 319.1 | 205 | 5 | 5 | 41.0 | 1 | 4 | 0.3 |
| 333.1 | 303 | 4 | 2 | 151.5 | 1 | 1 | 1.0 |
| 363.1 | 379 | 4 | 3 | 126.3 | 2 | 1 | 2.0 |
| 368.1 | 279 | 4 | 1 | 279.0 | 1 | 0 | 1.0 |
| 373.1 | 238 | 4 | 2 | 119.0 | 2 | 0 | 2.0 |
| 384.1 | 520 | 4 | 1 | 520.0 | 0 | 1 | 0.0 |
| 387.1 | 274 | 4 | 3 | 91.3 | 0 | 3 | 0.0 |
| 423.1 | 169 | 4 | 5 | 33.8 | 4 | 1 | 4.0 |
| 432.1 | 243 | 4 | 14 | 17.4 | 8 | 6 | 1.3 |
| 438.1 | 228 | 4 | 1 | 228.0 | 1 | 0 | 1.0 |
| 440.1 | 209 | 4 | 4 | 52.3 | 4 | 0 | 4.0 |
| 446.1 | 144 | 4 | 2 | 72.0 | 2 | 0 | 2.0 |
| 542.1 | 205 | 3 | 1 | 205.0 | 1 | 0 | 1.0 |
| 547.1 | 275 | 3 | 2 | 137.5 | 1 | 1 | 1.0 |
| 569.1 | 293 | 3 | 6 | 48.8 | 5 | 1 | 5.0 |
| 609.1 | 272 | 3 | 5 | 54.4 | 4 | 1 | 4.0 |
| 673.1 | 267 | 3 | 6 | 44.5 | 4 | 2 | 2.0 |
| 681.1 | 362 | 3 | 1 | 362.0 | 0 | 1 | 0.0 |
| 690.1 | 230 | 3 | 1 | 230.0 | 1 | 0 | 1.0 |
| 727.1 | 179 | 3 | 1 | 179.0 | 0 | 1 | 0.0 |
| 735.1 | 209 | 3 | 10 | 20.9 | 5 | 4 | 1.3 |
| 970.1 | 67 | 2 | 2 | 33.5 | 1 | 1 | 1.0 |
| 996.1 | 257 | 2 | 1 | 257.0 | 0 | 1 | 0.0 |
| 1040.1 | 269 | 2 | 3 | 89.7 | 2 | 1 | 2.0 |
| 1213.1 | 246 | 2 | 2 | 123.0 | 2 | 0 | 2.0 |
| 1333.1 | 332 | 2 | 3 | 110.7 | 3 | 0 | 3.0 |
| 1438.1 | 255 | 2 | 4 | 63.8 | 3 | 1 | 3.0 |
| 1540.1 | 252 | 2 | 1 | 252.0 | 0 | 1 | 0.0 |
