# Supplementary material for: Next-generation pyrosequencing of gonad transcriptomes in the polyploid lake sturgeon (Acipenser fulvescens): the relative merits of normalization and rarefaction in gene discovery
Source: BMC Genomics. 2009 Apr 29;10:203. doi: 10.1186/1471-2164-10-203 (PMC2688523; doi:10.1186/1471-2164-10-203)
Supplement: Additonal File 5 — Details of all SNPs in contigs with a signficant BLAST hit. Subset of contigs with a significant BLAST hit and at least one SNP. In other words, the SNPs described in this table are a subset of the 877 we identified, but include all of those associated with a particular gene. [file 1471-2164-10-203-S5.doc]

| Contig ID | Length | Depth | # SNPs | bp per SNP | Ts/Tv ratio | Top BLAST hit | GENBANK ID | Bit score | e-value |
| --- | --- | --- | --- | --- | --- | --- | --- | --- | --- |
| Contig0.1 | 1823 | 175 | 38 | 48.0 | 2.4 | Ac1147 | NP_001020173.1 | 202 | 1.00E-60 |
| Contig1.1 | 1201 | 142 | 45 | 26.7 | 1.8 | 2-oxoglutarate ferredoxin oxidoreductase | JC1348 | 148 | 5.00E-34 |
| Congit3.1 | 527 | 68 | 11 | 47.9 | 1.8 | Ribosomal S23 | NP_001103591.1 | 256 | 4.00E-67 |
| Contig8.1 | 704 | 43 | 14 | 50.3 | 1.8 | Putative transposase | CAC28060.1 | 99 | 1.00E-28 |
| Contig9.1 | 650 | 43 | 25 | 26.0 | 2.0 | ORF2 | BAA88337.1 | 152 | 2.00E-35 |
| Contig11.1 | 923 | 41 | 20 | 46.2 | 3.8 | Glutathione peroxidase 1 | NP_001007282.2 | 310 | 7.00E-83 |
| Contig12.1 | 1072 | 40 | 10 | 107.2 | 9.0 | Cytochrome c oxidase subunit I | NP_839826.1 | 522 | 1.00E-146 |
| Contig14.1 | 819 | 29 | 6 | 136.5 | 2.0 | Ribosomal protein S2 | ACH70782.1 | 468 | 1.00E-130 |
| Contig17.1 | 400 | 28 | 4 | 100.0 | 4.0 | 40S ribosomal protein S5 | ACI68342.1 | 145 | 8.00E-34 |
| Contig18.1 | 421 | 28 | 5 | 84.2 | 0.7 | CXXC finger 6 | XP_001072947.1 | 53 | 7.00E-06 |
| Contig19.1 | 379 | 27 | 8 | 47.4 | 0.5 | Ribosomal S20 | XP_001514106.1 | 181 | 2.00E-44 |
| Contig21.1 | 315 | 27 | 11 | 28.6 | 1.8 | 60S ribosomal protein L5 | CAH57700.1 | 82 | 1.00E-14 |
| Contig22.1 | 751 | 26 | 7 | 107.3 | 2.5 | Cytochrome c oxidase subunit II | NP_943620.1 | 369 | 1.00E-100 |
| Contig25.1 | 886 | 24 | 1 | 886.0 | 1.0 | Cytochrome c oxidase subunit III | NP_990956.1 | 427 | 1.00E-118 |
| Contig26.1 | 630 | 24 | 2 | 315.0 | 0.0 | ORF2 | AAC60281.1 | 89 | 1.00E-22 |
| Contig27.1 | 619 | 24 | 17 | 36.4 | 1.3 | RNA-directed protein | XP_001922760.1 | 97 | 4.00E-33 |
| Contig28.1 | 656 | 24 | 9 | 72.9 | 1.3 | Ribosomal L9 | ACH44170.1 | 148 | 3.00E-34 |
| Contig35.1 | 325 | 20 | 1 | 325.0 | 1.0 | Translation elongation factor 2 | AAL57757.1 | 61 | 2.00E-08 |
| Contig36.1 | 691 | 20 | 2 | 345.5 | 2.0 | Ubiquitin-protein | XP_002118344.1 | 53 | 2.00E-05 |
| Contig37.1 | 433 | 19 | 4 | 108.3 | 3.0 | Ribosomal protein L18 | AAP20219.1 | 221 | 1.00E-56 |
| Contig39.1 | 599 | 19 | 3 | 199.7 | 2.0 | SH2-B | XP_001054782.1 | 122 | 1.00E-26 |
| Contig40.1 | 738 | 19 | 11 | 67.1 | 1.2 | Transposase | ACH85309.1 | 55 | 1.00E-13 |
| Contig42.1 | 567 | 18 | 21 | 27.0 | 1.2 | Transposase | CAB51372.1 | 150 | 6.00E-35 |
| Contig44.1 | 413 | 17 | 10 | 41.3 | 9.0 | Ribosomal protein L26 | BAF98676.1 | 103 | 5.00E-21 |
| Contig46.1 | 619 | 17 | 9 | 68.8 | 8.0 | 60S acidic ribosomal protein P0 | Q90YX1 | 205 | 2.00E-51 |
| Contig50.1 | 526 | 16 | 6 | 87.7 | 5.0 | Ribosomal protein L23 | ABW04138.1| | 265 | 8.00E-70 |
| Contig51.1 | 408 | 16 | 2 | 204.0 | 1.0 | Collagen-like protein F | XP_001640108.1 | 48 | 3.00E-04 |
| Contig55.1 | 463 | 15 | 5 | 92.6 | 4.0 | S100 calcium binding | XP_001367941.1 | 57 | 4.00E-07 |
| Contig58.1 | 415 | 14 | 5 | 83.0 | 1.5 | Transposase | AAP49009.1 | 137 | 2.00E-31 |
| Contig60.1 | 293 | 14 | 3 | 97.7 | 2.0 | Macrophage migration inhibitory factor | ABG54274.1 | 112 | 9.00E-24 |
| Contig61.1 | 465 | 13 | 1 | 465.0 | 0.0 | Calmodulin Complex | 2VAY | 202 | 7.00E-51 |
| Contig63.1 | 345 | 13 | 4 | 86.3 | 3.0 | 60S ribosomal protein L22 | ACI69845.1 | 81 | 2.00E-14 |
| Contig65.1 | 553 | 13 | 7 | 79.0 | 0.8 | Endonuclease-reverse transcriptase | XP_001182458.1 | 80 | 6.00E-14 |
| Contig66.1 | 362 | 13 | 3 | 120.7 | 3.0 | Cytochrome c-oxidase family 3 | NP_001087454.1 | 91 | 4.00E-17 |
| Contig76.1 | 399 | 12 | 1 | 399.0 | 1.0 | Ribosomal protein L13a | BAF98661.1 | 127 | 3.00E-28 |
| Contig80.1 | 314 | 12 | 1 | 314.0 | 1.0 | Capg protein | AAH49461.1 | 137 | 3.00E-31 |
| Contig89.1 | 289 | 11 | 3 | 96.3 | 3.0 | 60S ribosomal protein L5 | CAH57700.1 | 61 | 2.00E-08 |
| Contig92.1 | 606 | 11 | 9 | 67.3 | 3.5 | Senescence-associated protein | BAB33421.1 | 74 | 7.00E-26 |
| Contig97.1 | 470 | 11 | 7 | 67.1 | 1.3 | RT-like superfamily | XP_001199386.1 | 129 | 6.00E-29 |
| Contig101.1 | 327 | 11 | 12 | 27.3 | 5.0 | Ribosomal protein L12 | ACH70859.1 | 147 | 4.00E-34 |
| Contig124.1 | 285 | 9 | 1 | 285.0 | 1.0 | mCG7861, isoform CRA_b | XP_001150801.1 | 46 | 8.00E-04 |
| Contig130.1 | 521 | 9 | 8 | 65.1 | 1.0 | Reverse transcriptase | BAD72127.1 | 69 | 1.00E-10 |
| Contig134.1 | 285 | 8 | 3 | 95.0 | 2.0 | Ribosomal protein L10a | BAF98657.1 | 145 | 1.00E-33 |
| Contig137.1 | 501 | 8 | 2 | 250.5 | 1.0 | Beta-actin | ACI23578.1 | 335 | 7.00E-91 |
| Contig138.1 | 412 | 8 | 4 | 103.0 | 3.0 | Ribosomal protein L32 | ACH44827.1 | 236 | 6.00E-61 |
| Contig140.1 | 263 | 8 | 3 | 87.7 | 2.0 | PREDICTED: hypothetical protein | XP_001232842.1 | 123 | 4.00E-27 |
| Contig153.1 | 437 | 8 | 7 | 62.4 | 6.0 | Transposase | CAB51372.1 | 99 | 5.00E-25 |
| Contig168.1 | 519 | 7 | 3 | 173.0 | 2.0 | ORF2 | BAA88337.1 | 100 | 7.00E-20 |
| Contig176.1 | 311 | 7 | 14 | 22.2 | 0.4 | Polyprotein | AAN12398.1 | 89 | 1.00E-16 |
| Contig191.1 | 353 | 7 | 2 | 176.5 | 2.0 | Macrophage migration inhibitory factor | ABG54276.1 | 111 | 2.00E-23 |
| Contig192.1 | 480 | 7 | 1 | 480.0 | 1.0 | unnamed protein product | CAF92285.1 | 59 | 1.00E-07 |
| Contig193.1 | 342 | 7 | 5 | 68.4 | 5.0 | Transcription factor | AAX30301.1 | 122 | 1.00E-26 |
| Contig215.1 | 467 | 6 | 2 | 233.5 | 1.0 | Large ribosomal subunit | XP_001094570.1 | 196 | 4.00E-49 |
| Contgi226.1 | 399 | 6 | 4 | 99.8 | 2.0 | CHK1 checkpoint-like protein | ABK29471.1 | 83 | 6.00E-15 |
| Contig239.1 | 250 | 6 | 2 | 125 | 1.0 | Reverse transcriptase-like protein | ABQ01988.1 | 77 | 6.00E-13 |
| Contig258.1 | 353 | 5 | 3 | 117.7 | 3.0 | Muscle fatty acid binding protein | NP_001117050.1 | 157 | 3.00E-37 |
| Contig278.1 | 485 | 5 | 3 | 161.7 | 0.0 | Tc1-like transporase | BAF37936.1 | 121 | 2.00E-26 |
| Contig279.1 | 323 | 5 | 1 | 323.0 | 1.0 | Fatty acid-binding protein | ACH43651.1 | 60 | 9.00E-09 |
| Contig373.1 | 238 | 4 | 2 | 119.0 | 2.0 | Endonuclease-reverse transcriptase | XP_001194331.1 | 74 | 3.00E-12 |
| Contig432.1 | 243 | 4 | 14 | 17.4 | 1.3 | LWamide neuropeptides precursor | Q25060 | 55 | 2.00E-06 |
